# Spatiotemporal Pattern of Bacillary Dysentery in China from 1990 to 2009: What Is the Driver Behind?

**DOI:** 10.1371/journal.pone.0104329

**Published:** 2014-08-05

**Authors:** Zhiwei Xu, Wenbiao Hu, Yewu Zhang, Xiaofeng Wang, Shilu Tong, Maigeng Zhou

**Affiliations:** 1 School of Public Health and Social Work, Queensland University of Technology, Brisbane, Australia; 2 Institute of Health and Biomedical Innovation, Queensland University of Technology, Brisbane, Australia; 3 Chinese Center for Disease Control and Prevention, Beijing, P. R. China; 4 National Center for Chronic and Noncommunicable Disease Control and Prevention, Chinese Center for Disease Control and Prevention, Beijing, P. R. China; Alberta Provincial Laboratory for Public Health/University of Alberta, Canada

## Abstract

**Background:**

Little is known about the spatiotemporal pattern of bacillary dysentery (BD) in China. This study assessed the geographic distribution and seasonality of BD in China over the past two decades.

**Methods:**

Data on monthly BD cases in 31 provinces of China from January 1990 to December 2009 obtained from Chinese Center for Disease Control and Prevention, and data on demographic and geographic factors, as well as climatic factors, were compiled. The spatial distributions of BD in the four periods across different provinces were mapped, and heat maps were created to present the seasonality of BD by geography. A cosinor function combined with Poisson regression was used to quantify the seasonal parameters of BD, and a regression analysis was conducted to identify the potential drivers of morbidity and seasonality of BD.

**Results:**

Although most regions of China have experienced considerable declines in BD morbidity over the past two decades, Beijing and Ningxia still had high BD morbidity in 2009. BD morbidity decreased more slowly in North-west China than other regions. BD in China mainly peaked from July to September, with heterogeneity in peak time between regions. Relative humidity was associated with BD morbidity and peak time, and latitude was the major predictor of BD amplitude.

**Conclusions:**

The transmission of BD was heterogeneous in China. Improved sanitation and hygiene in North-west China, and better access to clean water and food in the big floating population in some metropolises could be the focus of future preventive interventions against BD. BD control efforts should put more emphasis on those dry areas in summer.

## Introduction

Diarrhoea is an important cause of disease burden worldwide, particularly for resource-limited countries and for children under age of five [1], [2], [3], [4]. Bacillary dysentery (BD), a severe form of shigellosis, accounts for approximately five percent of diarrhoeal episodes [5], [6], and it contributes to over a million deaths annually worldwide [6]. Due to the increasing resistance of *shigella* to multiple antimicrobials [7], it is essential to develop effective strategies to control and prevent BD. China has made rapid economic progress in the past decades, and the morbidity and mortality of BD have been declining progressively [8], partially due to the substantially improved water supplies, sanitation and hygiene [9]. Nevertheless, it still shoulders a considerable burden of disease from BD [5].

Recognition of the geographic distribution of BD can guide future health resource allocation, and exploring BD seasonality and understanding the season-specific risk factors can shed new light on the development of future vaccination programs. So far, few data on the spatial pattern of BD in China are available, and existing literature offers limited information on the pattern and potential drivers of BD seasonality in the whole China. This study used data on the monthly BD cases in 31 provinces of China from 1990–2009 and attempted to: I) explore the geographic distribution of BD in China; II) characterize the seasonality of BD by geography in China; and III) identify the putative drivers of BD morbidity and seasonality.

## Materials and Methods

### Data collection

BD is a legally notifiable infectious disease in China. All clinical and hospital doctors are required to report cases of bacillary dysentery to local Center for Disease Control and Prevention (CDC). From 1990 to 2003, local CDCs reported the cases to China CDC once every month. From 2004 to 2009, the reporting system became internet based and doctors reported BD cases through Chinese Information System for Diseases Control and Prevention (CISDCP) [10]. Monthly data on BD cases in China from 1^st^ January 1990 through 31^st^ December 2009, nationally and provincially, were obtained from China CDC. The onset month and place of the cases were included in the data. Data on BD included both probable and lab-confirmed cases as defined by the national BD standard. Illness is characterized by tenesmus symptoms, pus stool, mucus stool, watery stool, or loose stool. Laboratory criteria for diagnosis are based on the positive isolation of *Shigella* bacteria.

Data on the demographic (population and per capita gross regional product (PGRP)) and geographic (latitude and longitude) characteristics and climate variables (monthly average mean temperature, relative humidity and rainfall) in different provinces were retrieved from China Statistical Yearbooks [11]. Ethical approval was obtained from the Human Research Ethics Committee of Queensland University of Technology (Australia) and the Research Institutional Review Board of Public Health of Shandong University (China) prior to the data being collected. Patient information was de-identified and thus no written informed consent was obtained.

### Statistical analysis

A seasonal decomposition analysis was conducted to assess whether there was a long-term trend and a distinct seasonality of BD in China [12]. Descriptive statistics of BD morbidity in China in four periods (1990–1994, 1995–1999, 2000–2004 and 2005–2009) were analysed to describe the dynamics of the disease. The spatial distributions of BD in the four periods across different provinces were also mapped.

In terms of BD seasonality, we created heat maps to present the peak and trough times of BD in each province [13], [14]. A cosinor function with Poisson regression was used to quantify the peak time, trough time and annual amplitude of BD [15]. For a better comparison between different provinces, the annual amplitude was expressed as a proportion of mean case counts. Regression tree analysis was conducted to identify the potential drivers of morbidity and seasonality of BD [16]. Visual maps were created using ArcGIS 9.3 (ESRI Inc., Redlands, CA, USA), and all other analyses were performed using R (version 2.15), with the “season” package (version 0.3–3) to conduct the cosinor analysis and “rpart” package to conduct the regression tree analysis.

## Results

**Figure 1:**
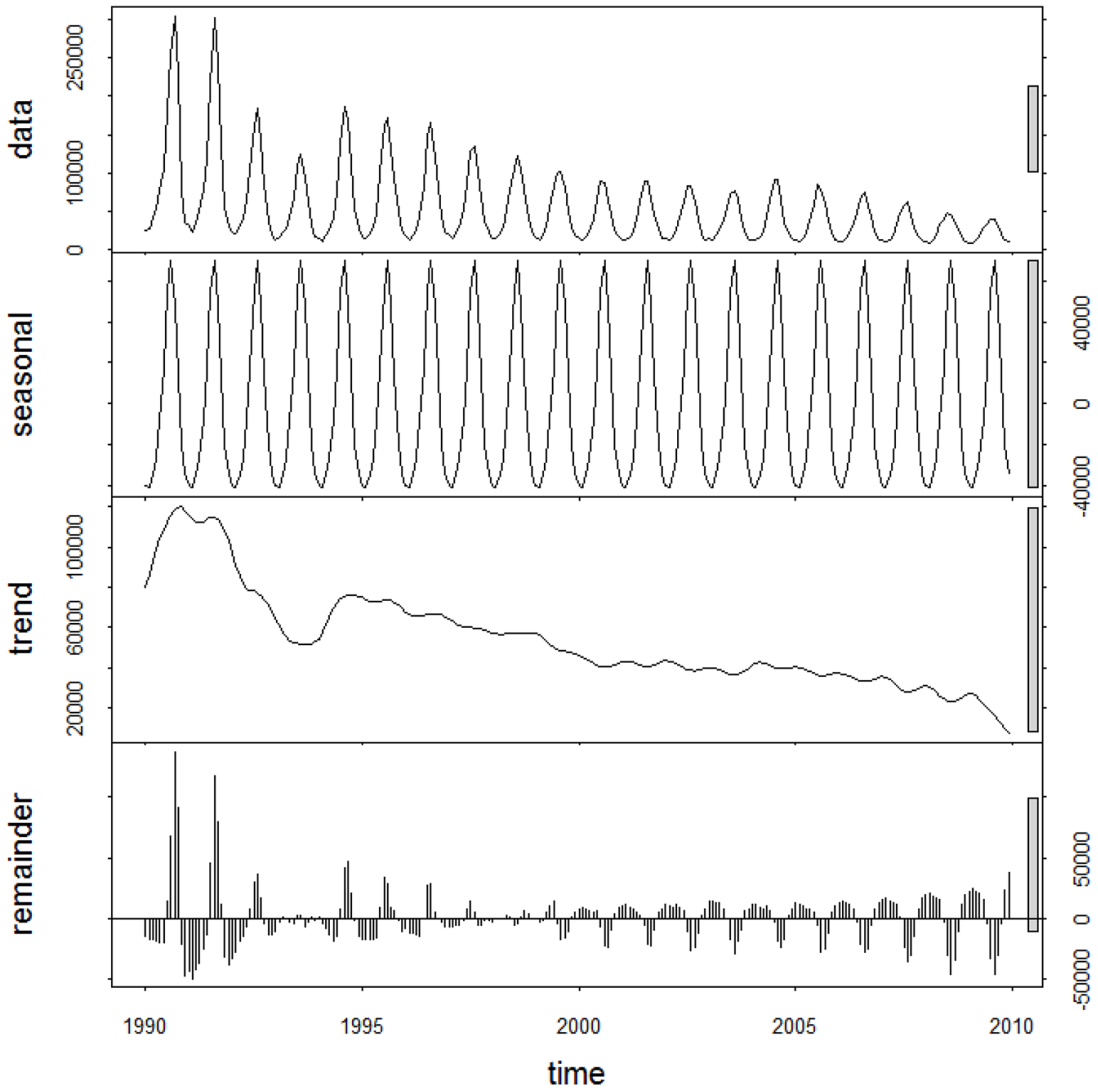
The decomposed distribution of monthly bacillary dysentery morbidity in China, from January 1990 to December 2009.

The summary statistics for BD morbidity in China over the four periods, including mean, maximum and minimum values, were presented in Table 1. The average values of BD morbidity over the four periods were 0.736, 0.493, 0.318 and 0.230 (per 10 000), respectively. The maximum and minimum values of BD morbidity also experienced a gradual decrease across the four periods. Figure 1 presented the 20-year decomposed distribution of BD cases, revealing a decreasing long-term trend and a distinct seasonality.

**Table 1:** Summary statistics for monthly incidence of bacillary dysentery (1/10 000) in China, from 1990–2009.

Time	Mean	Standard deviation	Minimum	Maximum
Morbidity (1/10 000)				
Period 1 (1990–1994)	0.736	0.654	0.100	2.667
Period 2 (1995–1999)	0.493	0.370	0.099	1.417
Period 3 (2000–2004)	0.318	0.207	0.095	0.704
Period 4 (2005–2009)	0.230	0.152	0.070	0.652

**Figure 2:**
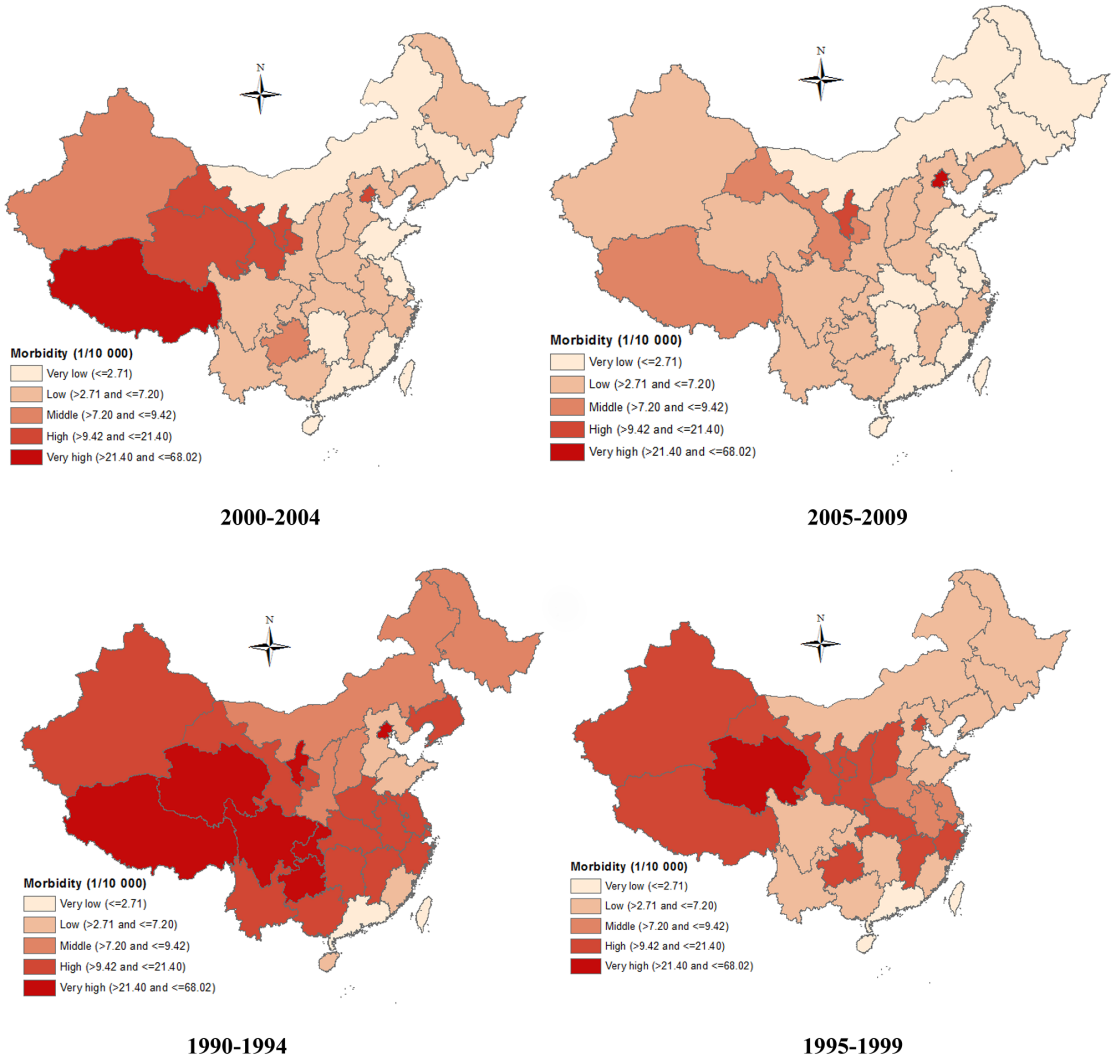
The spatial patterns of bacillary dysentery morbidity across four periods in China, from 1990–2009.

Figure 2 showed the spatial patterns of BD morbidity over the four periods, illustrating that BD morbidity experienced a sharp decrease in most regions during the 20 years. In North China (Beijing, Tianjin, Hebei, Shanxi and Inner Mongolia), a significant decrease in BD morbidity was observed. In North-east China (Liaoning, Jilin, and Heilongjiang), East China (Jiangsu, Zhejiang, Anhui, Fujian, Jiangxi, and Shandong) and Central and South China (Henan, Hubei, Hunan, Guangdong, Guangxi and Hainan), gradual declines in BD morbidity occurred in the 20 years. In South-west China (Sichuan, Guizhou, Yunnan, Chongqing, Tibet), we found BD morbidity declined sharply (from very high to very low) in Sichuan and Yunnan from the first period (1990–1994) to the second period (1995–1999), while BD morbidity in Tibet and Guizhou remained quite high in the first two periods (1990–1999). BD morbidity in most provinces of North-west China (Shaanxi, Gansu, Qinghai and Xinjiang) did not decrease much until the last period (2005–2009), and BD morbidity in Ningxia was consistently high during the 20 years.

**Figure 3:**
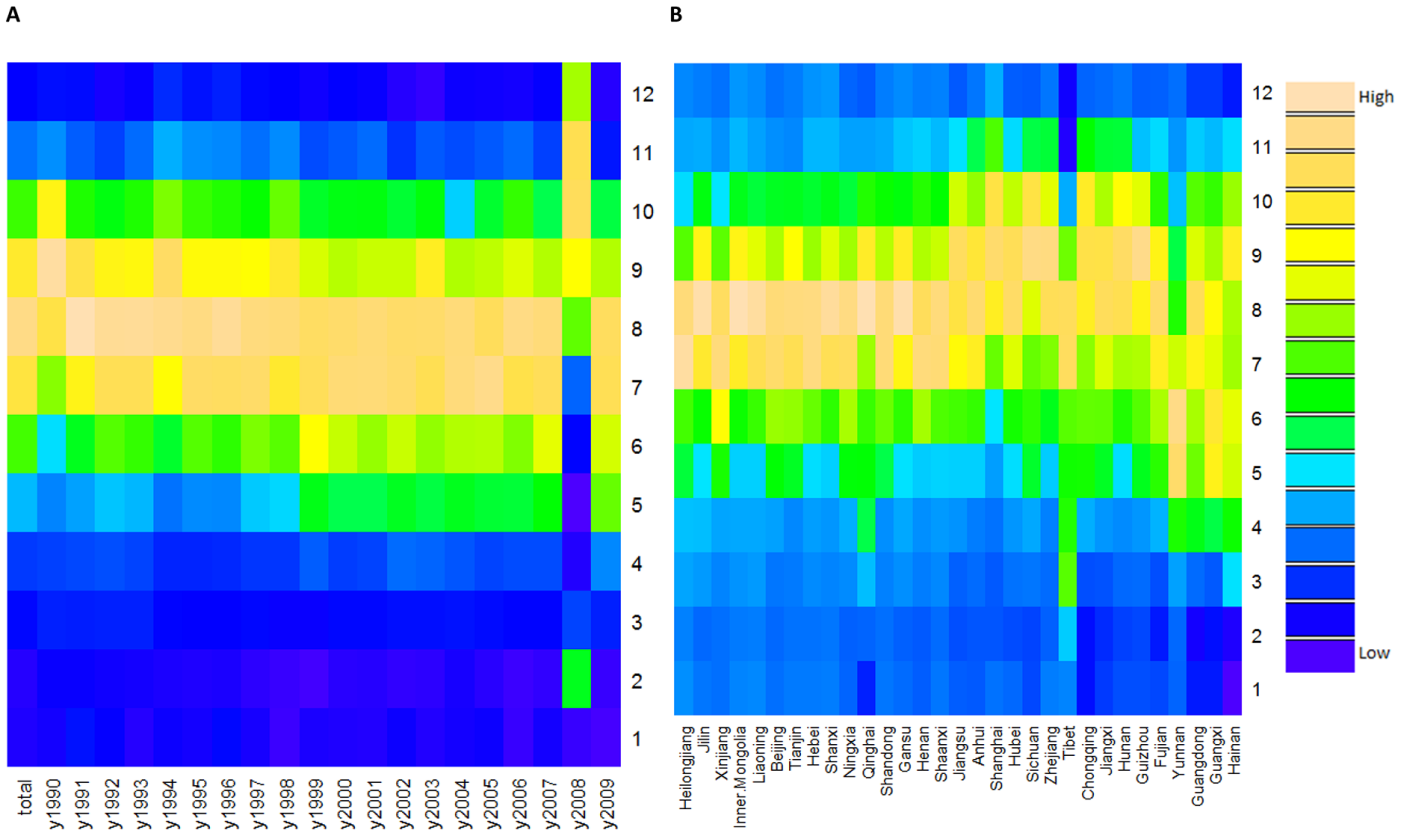
Heat maps of bacillary dysentery epidemiology data in the whole China (A) and 31 provinces of China (B), from 1990 to 2009.

**Figure 4:**
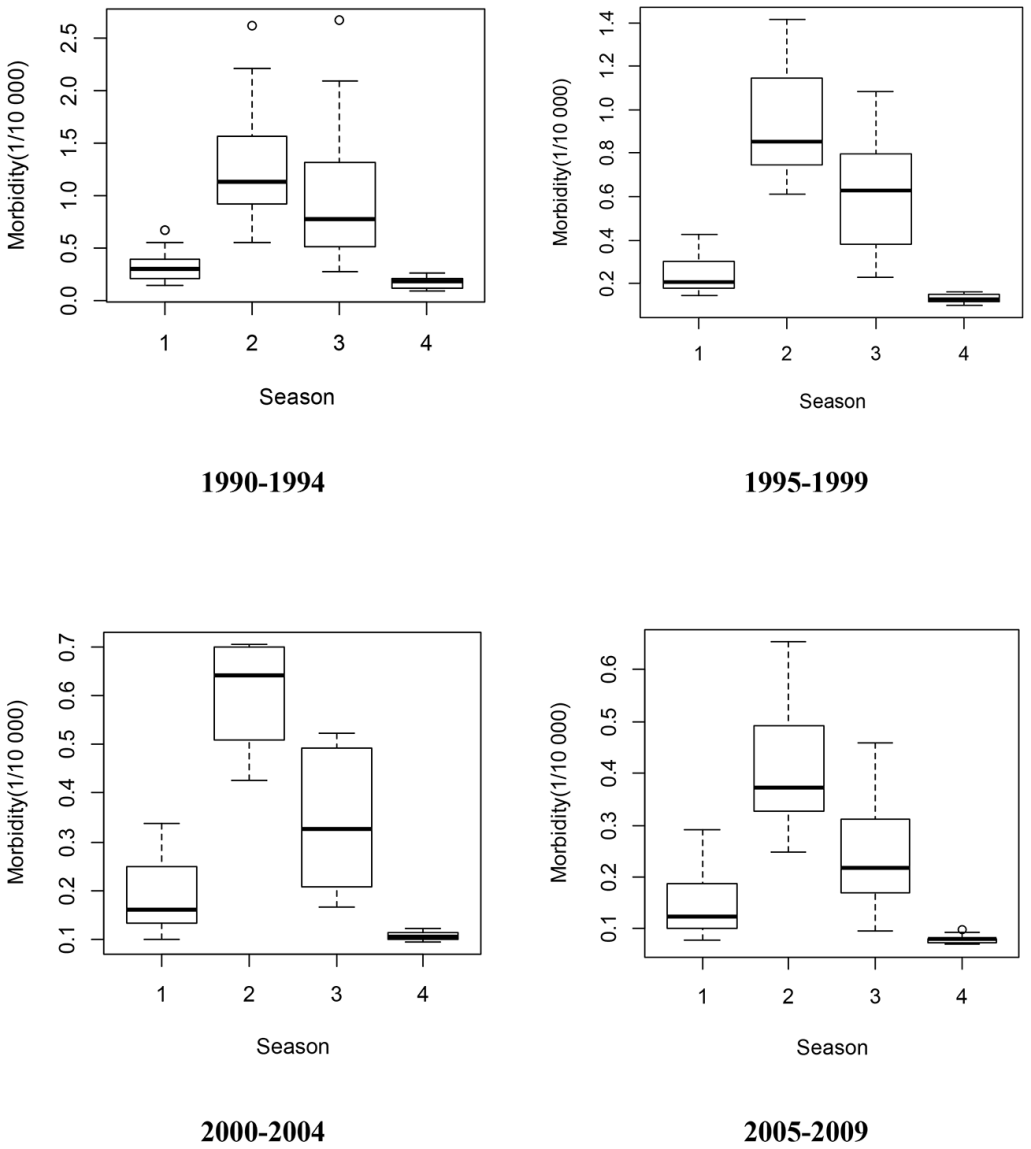
The distributions of bacillary dysentery morbidity across four seasons in China, from 1990 to 2009 (1 = spring, 2 = summer, 3 = autumn, 4 = winter).

**Figure 5:**
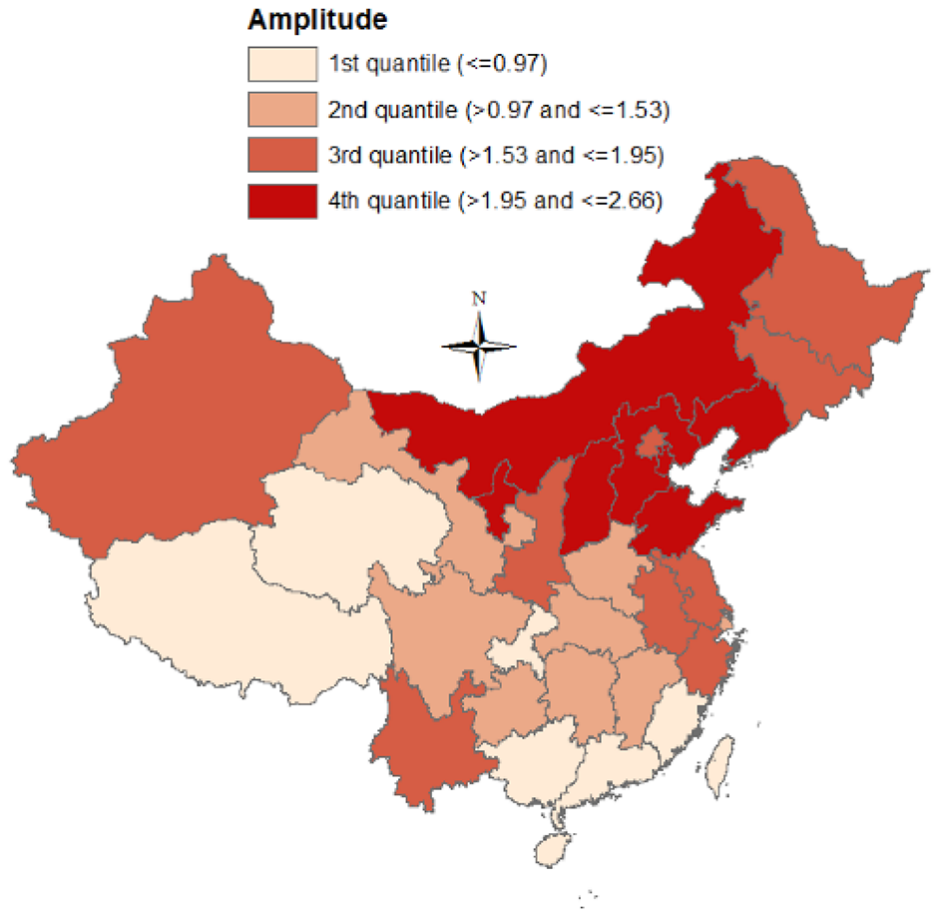
The relative amplitude of bacillary dysentery seasonality in China.

Figure 3A revealed a relatively constant seasonality in China from 1990–2009, indicating that BD was more likely to peak in summer (July–September), even though there were two exceptions (1990 and 2008). Figure 3B showed the seasonality of BD by geography (listed by latitude), revealing that BD appeared to peak from July–September at high latitudes, and peak from July–October at intermediate latitudes. At low latitudes, BD peak time varied greatly between provinces (especially Yunnan and Guangxi), from May–October. Summer and autumn were the most pandemic seasons (Figure 4), and the morbidity difference between summer and autumn during 2000–2004 was higher than other three periods. Figure 5 presented the spatial pattern of BD amplitude, showing that higher latitudes (Northern China) were more likely to have greater amplitudes.

**Figure 6:**
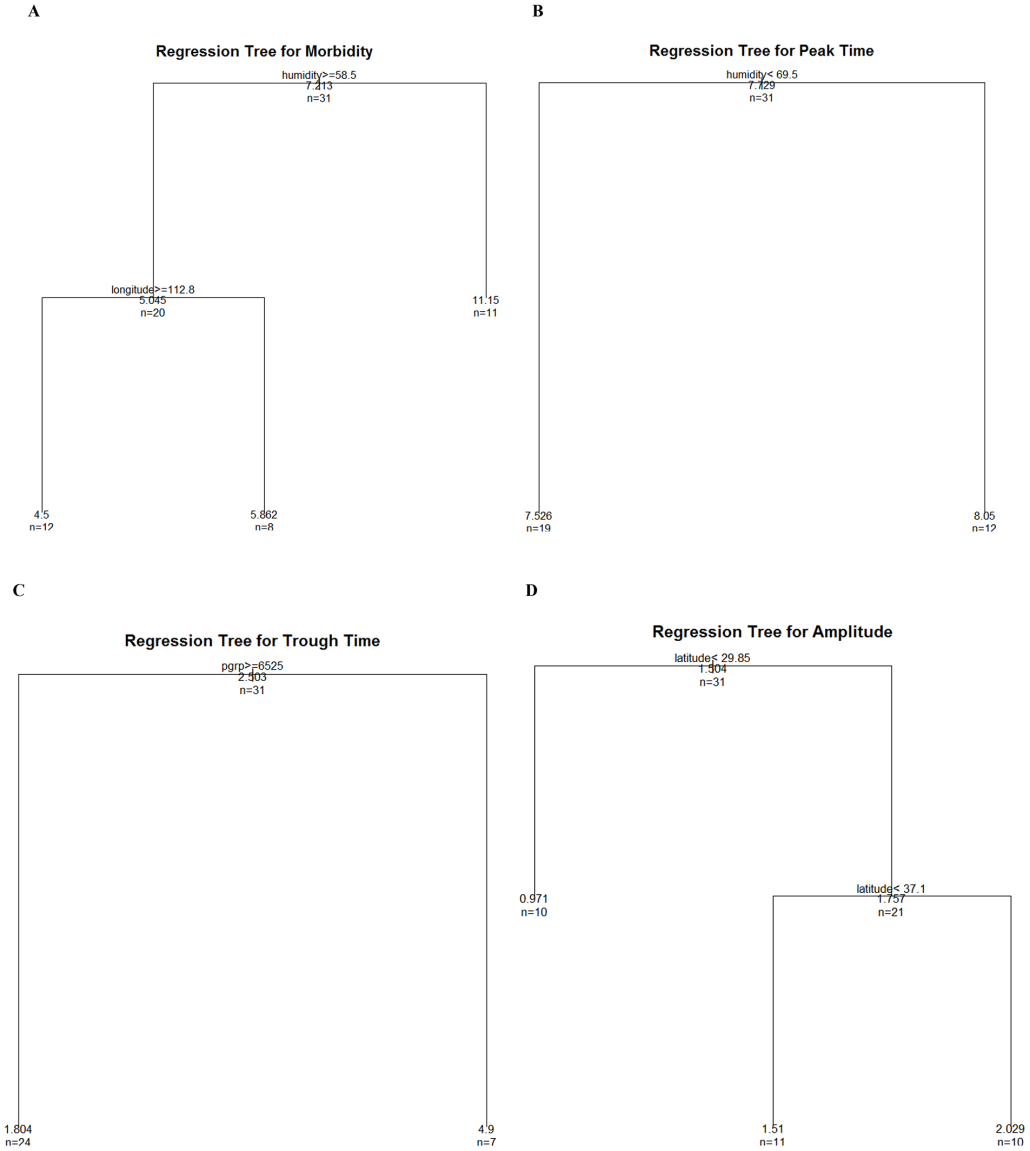
Predictors of bacillary dysentery morbidity and seasonality in China.

The summary statistics for the demographic and geographic characteristics, climatic factors, and BD seasonal parameters of the 31 provinces in China, which were included in the regression tree model, were presented in Table 2. The potential drivers of BD morbidity and seasonality were unveiled in Figure 6. Relative humidity and longitude were significantly associated with BD morbidity. Provinces with dry (monthly average relative humidity<58.5%) climate shouldered greater BD burdens. Relative humidity was also associated with BD peak time, with provinces with monthly average relative humidity <69.5% peaking earlier. PGRP was associated with BD trough time, and provinces with higher PGRP reached trough time earlier than those with lower PGRP. Latitude was the primary predictor of BD amplitude, and the two latitudinal thresholds were 29.85°N and 37.1°N.

**Table 2:** Summary statistics for geographic characteristics, climatic factors, and bacillary dysentery seasonal parameters of the 31 provinces in China.

Region	Latitude	Longitude	PGRP*	Temperature (°C)	Humidity (%)	Rainfall (mm)	Morbidity	Peak	Trough	Amplitude
Anhui	31.8	117.5	8219.8	17	75	33	6	8.1	2.1	1.6
Beijing	39.9	116.4	23034.3	14	52	23	20.4	7.5	1.5	1.95
Chongqing	29.6	106.6	6431.1	19	81	35	6.3	8.2	2.2	0.84
Fujian	25.3	118.8	13435.4	21	69	33	1.7	7.7	1.7	0.97
Gansu	35.6	104.7	4861.9	10	53	27	10.4	7.9	1.9	1.53
Guangdong	22.9	113.4	14628.1	23	71	33	1.7	7.4	1.4	0.62
Guangxi	22.9	108.4	5512.2	22	76	36	4.4	7.1	1.1	0.91
Guizhou	27.4	106.8	4196.7	15	75	34	10.7	8.1	2.1	1.27
Hainan	19.6	110.1	7865.7	24	80	35	2.3	7.2	1.2	0.34
Hebei	38.1	115.8	9240	15	57	25	4.4	7.6	1.6	2.05
Heilongjiang	46.1	126.2	9978.5	6	61	27	4	7.4	1.4	1.9
Henan	34.7	113.1	8197	16	59	28	5.1	7.7	1.7	1.46
Hubei	30.9	112.6	8131.3	18	70	33	6.3	8.2	2.2	1.41
Hunan	27.4	113	7061.7	19	72	33	3.5	8.3	2.3	1.35
Jiangsu	32.9	118.6	14190.9	17	71	33	4.7	8.2	2.3	1.62
Jiangxi	28.2	115.3	6619.6	19	69	33	5.7	8.1	2.1	1.32
Jilin	44.1	125.4	9440	7	59	27	4.6	7.8	1.8	1.54
Liaoning	40.7	122.6	15240.3	8	66	26	5.7	7.7	1.7	2.13
Mongolia	40.8	110.8	9562.3	8	47	21	3.5	7.9	1.9	2.04
Ningxia	37.6	106	6876.5	10	50	23	14.5	7.4	1.4	1.96
Qinghai	36.6	101.8	6890.9	6	57	24	13	7.7	1.7	0.75
Shaanxi	34.3	108.8	7518.6	15	62	31	5.6	7.7	1.7	1.77
Shandong	36.3	118.4	6100.4	15	57	28	3.6	7.7	1.7	2.2
Shanghai	31.3	121.5	20782.4	18	70	31	5.1	8.9	2.9	1.4
Shanxi	37.8	112.8	5928.6	11	54	24	5.6	7.8	1.8	2.11
Sichuan	30.2	104	8691.1	17	75	30	5.7	8.4	2.4	1.2
Tianjin	39.2	117.2	20782.4	13	58	26	14.5	7.6	1.6	2.66
Tibet	29.7	91.1	5928.6	10	34	35	23.9	6.5	12.5	0.43
Xinjiang	43.8	87.6	8691.1	8	55	25	8.9	7.2	1.2	1.95
Yunnan	24.8	103	5383.2	16	69	31	5.6	6.1	12.1	1.66
Zhejiang	30	120.4	16457.4	18	71	30	6.2	8.5	2.5	1.67

*PGRP: per capita gross regional product.

## Discussion

This study examined the spatiotemporal pattern of BD in China from 1990 to 2009. China has experienced a significant decrease in BD morbidity over the past two decades, although the decrease was not uniform between regions and Beijing and Ningxia still shouldered heavy BD burdens in 2009. In North-west China, BD morbidity declined slowly, compared with East and Central China. The majority of BD cases occurred between July and October, even though heterogeneity in BD peak and trough times existed between regions. Relative humidity was found predictive of BD morbidity and peak time. PGRP helped distinguish BD trough time, and latitude was the major driver of BD amplitude in China.

BD risk map in China has been shrinking in the past two decades, due largely to the fast progressing economy, much improved hygiene and better access to sanitary water and food [9]. However, BD still remains the first or second dominating infectious disease in many regions [17], [18]. In this study, we found that BD morbidity was unacceptably high in Beijing and Ningxia throughout the whole study period (1990–2009), indicating that not only regions with low economic development had high BD infection. Previous studies indicated that the high BD morbidity in Beijing can be attributable to the relatively low under-reporting rate [17], the poor street food sanitation in some districts [19], and the emergence of *shigellosis* strains resistant to multiple antimicrobials resulted from overuse of antibiotics [17], [19], [20]. In China, over the past decades, millions of people in rural areas, especially physical workers (e.g., builders), migrate to metropolis like Beijing, working for a better salary. Majority of the floating population live in areas with poor access to clean water, which may also cause the high BD morbidity in Beijing [20]. Prior research offers limited information on BD in Ningxia. Wang et al. analysed BD in Ningxia from 1958 to 1997 and found that students and farmers, and people living in places with close proximity to river had increased risk of BD [18]. People living nearby rivers are more likely to use river water for bathing, washing, and cleaning of eating, which might expose them more to sewage compared with people who live far away from river [21].

In this study, we found BD predominantly peaked between July and October in China, corresponding to the BD peak time in Vietnam [22]. Geographical diversity in BD peak time was also found, with BD at high latitudes (northern China) mainly peaking between July and August, and BD at low latitudes (southern China) peaking between May to October. Existing science offers limited information on the drivers of special BD seasonality in Yunnan and Guangxi, and we speculated that it may be because of the different monsoon seasons in these regions [23], [24]. The motive of this study was to assist BD surveillance system and help develop future early warning system. In light of the diversity of BD peak time by geography, people living in northern China should be made aware of the high BD risk from July to August and BD surveillance in southern China should be extensively strengthened. Greater BD amplitude of seasonality at higher latitudes was also detected in this study, indicating that some latitude-related factors, such as the reduced sunlight and its possible effect on Vitamin D level [25], may contribute to the BD seasonality in China [26].

The regression tree approach we used in this study avoided the multi-collinearity among different meteorological factors (temperature, rainfall and relative humidity), and the results suggested that relative humidity may impact BD morbidity, echoing to prior studies examining the relationship between climate factors and BD [27], [28]. The underlying reason may be because humidity affects BD pathogens [27] and normally high relative humidity comes with high rainfall which reduces contaminated food and water [28]. In terms of the putative predictors of BD seasonality, we found relative humidity and PGRP were associated with BD peak time and trough time, respectively, and latitude was the primary predictor of BD amplitude, unveiling that BD seasonality may result from the interaction between climate, socioeconomic factors (which is associated with population's access to clean water and food) and population immunity to specific pathogens.

This study is, to our best knowledge, the first study to look at spatial pattern and seasonality in the whole China. The risk areas and peak and trough times we identified will assist BD surveillance. Several limitations of this study should be acknowledged. First, under-reporting of BD morbidity might result in information bias to some extent due to the use of surveillance data. Second, the provincial-level data we used are not ideal in identifying the small-scale BD high risk areas. Third, access to health care may vary across provinces, and the difference in reporting that may exist across provinces, which may result in information bias.

## Conclusions

China has undergone a substantial decrease in BD morbidity from 1990–2009, but the progress is heterogeneous between regions. BD in China peaked mainly between July and October, but the peak month varied by place and time. People in western China, especially those living in regions with low socioeconomic status, were more likely to be attacked by BD. BD control and prevention interventions should focus more on the regions with large floating populations or less-developed economy, and BD surveillance in southern China needs to be strengthened.

## References

[1] Bhutta ZA, Das JK, Walker N, Rizvi A, Campbell H, et al (2013) Interventions to address deaths from childhood pneumonia and diarrhoea equitably: what works and at what cost? Lancet 381: 1417–1429. 23582723 10.1016/S0140-6736(13)60648-0

[2] Chopra M, Mason E, Borrazzo J, Campbell H, Rudan I, et al (2013) Ending of preventable deaths from pneumonia and diarrhoea: an achievable goal. Lancet 381: 1499–1506. 23582721 10.1016/S0140-6736(13)60319-0

[3] Gill CJ, Young M, Schroder K, Carvajal-Velez L, McNabb M, et al (2013) Bottlenecks, barriers, and solutions: results from multicountry consultations focused on reduction of childhood pneumonia and diarrhoea deaths. Lancet 381: 1487–1498. 23582720 10.1016/S0140-6736(13)60314-1

[4] Walker CLF, Rudan I, Liu L, Nair H, Theodoratou E, et al (2013) Global burden of childhood pneumonia and diarrhoea. Lancet 381: 1405–1416. 23582727 10.1016/S0140-6736(13)60222-6 PMC7159282

[5] Von Seidlein L, Kim DR, Ali M, Lee H, Wang X, et al (2006) A multicentre study of shigella diarrhoea in six Asian countries: disease burden, clinical manifestations, and microbiology. PLoS Med 3: e353. 16968124 10.1371/journal.pmed.0030353 PMC1564174

[6] Kotloff K, Winickoff J, Ivanoff B, Clemens J, Swerdlow D, et al (1999) Global burden of Shigella infections: implications for vaccine development and implementation of control strategies. Bull World Health Organ 77: 651–666. 10516787 PMC2557719

[7] Gu B, Ke X, Pan S, Cao Y, Zhuang L, et al (2013) Prevalence and trends of aminoglycoside resistance in Shigella worldwide, 1999–2010. J Biomed Res 27: 103–115. 23554801 10.7555/JBR.27.20120125 PMC3602868

[8] Wang XY, Tao F, Xiao D, Lee H, Deen J, et al (2006) Trend and disease burden of bacillary dysentery in China (1991–2000). Bull World Health Organ 84: 561–568. 16878230 10.2471/blt.05.023853 PMC2627389

[9] Wang XY, Du L, Von SL, Xu ZY, Zhang YL, et al (2005) Occurrence of shigellosis in the young and elderly in rural China: results of a 12-month population-based surveillance study. Am J Trop Med Hyg 73: 416–422. 16103614

[10] Yang W, Li Z, Lan Y, Wang J, Ma J, et al (2011) A nationwide web-based automated system for early outbreak detection and rapid response in China. Western Pac Surveill Response J 2: 10–15. 10.5365/WPSAR.2010.1.1.009 PMC3729055 23908878

[11] China National Bureau of Statistics (2012) China Statistic Yearbook.

[12] Xu Z, Hu W, Su H, Turner LR, Ye X, et al (2014) Extreme temperatures and paediatric emergency department admissions. J Epidemiol Community Health 68: 304–311. 24272920 10.1136/jech-2013-202725

[13] Xing W, Liao Q, Viboud C, Zhang J, Sun J, et al (2014) Hand, foot, and mouth disease in China, 2008–12: an epidemiological study. Lancet Infect Dis 14: 308–318. 24485991 10.1016/S1473-3099(13)70342-6 PMC4035015

[14] Yu H, Alonso WJ, Feng L, Tan Y, Shu Y, et al (2013) Characterization of Regional Influenza Seasonality Patterns in China and Implications for Vaccination Strategies: Spatio-Temporal Modeling of Surveillance Data. PLoS Med 10: e1001552. 24348203 10.1371/journal.pmed.1001552 PMC3864611

[15] Barnett AG, Dobson AJ (2010) Analysing Seasonal Health Data: Springer.

[16] Hu W, Mengersen K, McMichael A, Tong S (2008) Temperature, air pollution and total mortality during summers in Sydney, 1994–2004. Int J Biometeorol 52: 689–696. 18506490 10.1007/s00484-008-0161-8

[17] Gao T, Liu G, Li X, Jia L, Liu Y, et al (2007) Analysis about epidemic situation of dysentery near upon fourteen years in Beijing. Chinese Journal of Preventive Medicine 41: 54–57 (Article in Chinese). 17484213

[18] Wang X, Yan L, Ren J, Hou Z, Li X (1999) The analysis of epidemiological features of bacillary dysentery in Ningxia, from 1958 to 1997. Chinese Journal of Epidemiology 20: 62 (Article in Chinese).

[19] Pei H, Tang Y (1998) Epidemiological analysis of dysentery in Beijing (1990∼1997). Disease Survilliance 13: 408–413 (Article in Chinese).

[20] Sun P, Qin A, Pu Y (2008) An analysis of epidemiological features of bacillary dysentery in Haidian District of Beijing during 1999–2005. Capital Journal of Public Health 2: 55–57 (Article in Chinese).

[21] Kim DR, Ali M, Thiem VD, Park J-K, von Seidlein L, et al (2008) Geographic analysis of shigellosis in Vietnam. Health & Place 14: 755–767. 18296100 10.1016/j.healthplace.2007.12.003

[22] Kelly-Hope L, Alonso W, Thiem V, Canh D, Anh D, et al (2008) Temporal trends and climatic factors associated with bacterial enteric diseases in Vietnam, 1991–2001. Environ Health Perspect 116: 7–12. 18197292 10.1289/ehp.9658 PMC2199291

[23] Li Y, Lin M, Liang D (2009) Epidemiological analysis of bacillary dysentery in Guangxi in 1998∼2007. China Tropical Medicine 9: 318–319.

[24] Wang J-F, Wang Y, Zhang J, Christakos G, Sun JL, et al (2013) Spatiotemporal transmission and determinants of typhoid and paratyphoid eever in Hongta District, Yunnan Province, China. PLoS Negl Trop Dis 7: e2112. 23516653 10.1371/journal.pntd.0002112 PMC3597484

[25] Kull M, Kallikorm R, Tamm A, Lember M (2009) Seasonal variance of 25-(OH) vitamin D in the general population of Estonia, a Northern European country. BMC Public Health 9: 22. 19152676 10.1186/1471-2458-9-22 PMC2632995

[26] Thornton KA, Marín C, Mora-Plazas M, Villamor E (2013) Vitamin D Deficiency Associated With Increased Incidence of Gastrointestinal and Ear Infections in School-age Children. Pediatr Infect Dis J 32: 585–593. 23340562 10.1097/INF.0b013e3182868989

[27] Huang D, Guan P, Guo J, Wang P, Zhou B (2008) Investigating the effects of climate variations on bacillary dysentery incidence in northeast China using ridge regression and hierarchical cluster analysis. BMC Infectious Diseases 8: 130. 18816415 10.1186/1471-2334-8-130 PMC2569063

[28] Li Z, Wang L, Sun W, Hou X, Yang H, et al (2013) Identifying high-risk areas of bacillary dysentery and associated meteorological factors in Wuhan, China. Sci Rep 3: 3239. 24257434 10.1038/srep03239 PMC3836034

